# CAC1 knockdown reverses drug resistance through the downregulation of P-gp and MRP-1 expression in colorectal cancer

**DOI:** 10.1371/journal.pone.0222035

**Published:** 2019-09-10

**Authors:** Nanzheng Chen, Ying Kong, Yunhua Wu, Qi Gao, Junke Fu, Xuejun Sun, Qianqian Geng

**Affiliations:** 1 The thoracic surgery department of the First affiliated hospital of Xi’an Jiaotong University, Xi’an, China; 2 The general surgery department of the First affiliated hospital of Xi’an Jiaotong University, Xi’an, China; 3 The medical oncology department of the First affiliated hospital of Xi’an Jiaotong University, Xi’an, China; 4 The nuclear medicine department of the First affiliated hospital of Xi’an Jiaotong University, Xi’an, China; Universitat des Saarlandes, GERMANY

## Abstract

CDK2-associated cullin domain 1 (CAC1) is as a novel cell cycle regulator widely expressed in colorectal cancer (CRC). However, its expression and function in drug resistant CRC cells remains elusive. Therefore, the present study aimed to assess the biochemical function and relevance of CAC1 in drug resistant CRC cells, and detect the potential mechanism. For this purpose, a total of 83 CRC cases were collected for the immunohistochemical analysis of CAC1 expression. Functional studies (stable transfection, flow cytometry, colony formation, and invasion and migration assays) were performed in SW480, LoVo and their corresponding 5-FU resistant cells. In addition, a nude mice xenograft model was established for further observation *in vivo*. In the present study, CAC1 protein expression was higher in CRC tissues than that in normal tissues (*P*<0.05). Furthermore, CAC1 protein expression was higher in SW480/5-FU cells than in SW480 cells. CAC1 knockdown arrested 5-FU resistant cells at the G1/S phase and increased the sensitivity of 5-FU resistant cells to 5-FU by inducing apoptosis. In addition, CAC1 reduced the invasive and migration ability of SW480/5-FU and LoVo/5-FU cells *in vitro*, and reduced their tumorigenicity and metastatic ability *in vivo*. Finally, CAC1 knockdown resulted in decreased P-glycoprotein and MRP-1 protein expression. Based on these results, it can be concluded that CAC1 plays an important role in the occurrence and promotion of drug resistance in CRC. Therefore, the knockdown of CAC1 may be considered as a new strategy for the development of CRC drug resistance treatments in the future.

## Introduction

Colorectal cancer (CRC) is the third most common cancer worldwide and the second leading cause of cancer-related mortality [1, 2]. The prognosis of CRC patients has slowly but steadily improved during the past decades [3]. The five-year survival rate has reached almost 65% in high-income countries [3]. However, in some countries, including China, the five-year survival rate remains <50% [4–6]. It has been shown that specific lifestyle factors, such as physical activity [7] and dietary structure (rich in fruit and vegetables) [8], together with early diagnostics and treatment improvements, such as endoscopy with the removal of precancerous lesions [9], have sharply reduced CRC incidence and mortality. Nevertheless, regardless of the archived progress, the five-year survival rate continues to be pessimistic due to cancer recurrence and drug resistance. For recurrent or metastatic CRC patients, chemotherapy remains as the main choice of treatment [10]. Therefore, determining new molecular targets that could be targeted to reverse drug resistance is of great significance, and is urgently needed.

CDK2-associated cullin domain 1 (CAC1) is mapped to chromosome 10 in humans (10q35-36, Gene Bank accession number AY743663) [11], and is classified as a cullin domain-containing protein and a member of the cullin family of E3 ubiquitin ligases [12]. It has been shown that CAC1 promotes cell cycle progression and activates cyclin-dependent kinase 2 (CDK2) kinase activity *via* multiple extracellular signals, including growth factors, mitogens, deoxycholic acid (DCA), curcumin, and even chemotherapy drugs [13, 14]. In addition to the already reported CAC1 role in cell cycle regulation in CRC cell line HCT-8 and gastric cell line AGS, several recent studies have shown that CAC1 acts as a corepressor of retinoic acid receptor-α (RARα) [15], and is involved in ERα regulation by binding to it and repressing its transcriptional activity [16]. In the hippocampus of Alzheimer disease patients, CAC1 has been found to be downregulated, and act as a protective factor against H_2_O_2_ and Aβ toxicity [17]. However, the biochemical function and relevance of CAC1 in drug resistance remains unexamined.

CDKs control the cell cycle, apoptosis and RNA transcription. The study conducted by Guo *et al*. [18] revealed that CDK2 protein was remarkably changed in the 5-fluorouracil (5-FU) resistant colorectal cell line, when compared with the parental cell line, indicating that cell cycle perturbation is involved in acquiring 5-FU resistance. Kaliszczak *et al*. [19] further reported that CDK activity affected ABC transporter-induced drug resistance. Nevertheless, in addition to being a CDK2 activator, the role of CAC1 in drug resistance has not been fully clarified.

In the present study, the hypothesis was that CAC1 may be involved in drug resistance in CRC by affecting the ABC transporter. In order to examine this hypothesis, in human CRC cell line SW480, LoVo, and their corresponding 5-FU resistant cell lines, shRNA stable transfection was used to knockdown CAC1, and the effects were further examined *in vitro* and *in vivo*. In addition, the potential underlying mechanism of the CAC1 function was further investigated to identify new possible targets for the treatment of drug resistance in CRC.

## Materials and methods

### Patients and samples

A total of 83 CRC patients, who underwent surgery in the First Affiliated Hospital of Xi’an Jiaotong University between March 2008 and March 2009, were recruited into the present study. These patients comprised of 55 males and 28 females, and the age of these patients was between 18 and 79 years, with a median age of 52 years. Among these 83 patients, 46 patients had colon cancer, while 37 patients had rectal cancer. The tumor stage of these patients were assessed based on the American Joint Committee on Cancer (AJCC)-TNM staging system [20]: 62 patients were stage I-II, and 21 patients were stage III-IV. Grading was performed according to the World Health Organization (WHO) criteria [21]: five tumors were well-differentiated, 60 tumors were moderately differentiated, and 18 tumors were poorly differentiated. All patients included in the present study did not receive any chemotherapy or radiation prior to surgery. The clinicopathological features of the 83 CRC cases are presented in Table 1. In addition to tumor tissues, normal colon/rectal tissues that were more than 5 cm away from the tumor margin were collected and used as controls. The present study was approved by the Ethics Committee of the First Affiliated Hospital of Xi’an Jiaotong University, and followed the guidelines of the Declaration of Helsinki. All patients who met the inclusion criteria provided a written informed consent.

**Table 1:** Clinicopathological features of the 83 CRC patients and their tumors.

Clinicopathological feature	*n*	CAC1 protein expression		*X*^*2*^	*P*
		Negative	Positive		
**Age**					
<60	49	10	39	0.098	0.492
≥60	34	6	28		
**Gender**					
Male	55	9	46	0.899	0.255
Female	28	7	21		
**Tumor location**					
Colon	46	7	39	1.093	0.222
Rectum	37	9	28		
Tumor grade*					
Well -moderately	65	12	53	0.128	0.476
Poorly	18	4	14		
**Lymph node metastasis**					
Yes	34	12	22	**9.495**	**0.003**
No	49	4	45		
**TNM staging**					
I- II	62	8	54	**6.398**	**0.017**
III- IV	21	8	13		

*According to the WHO classification of CRC

### Immunohistochemistry

For the immunohistochemistry analysis, all tissue samples were formalin-fixed, paraffin-embedded and cut into 4-μm-thick sections. In brief, the tissue sections were deparafinized in xylene, rehydrated in graded ethanol, and incubated with primary rabbit polyclonal antibody against CAC1 (dilution 1:100; GeneTex, Texas, USA; Cat no. GTX118514) for two hours at room temperature. Next, the slides were incubated with the secondary antibody (dilution 1:2,000; Pioneer Biotechnology, Shaanxi, China; Cat No. 31460) at room temperature for 30 minutes and washed in tris-buffered saline, and 3-amino-9-ethylcarbazole was used as the chromogen. Finally, the slides were counterstained with hematoxylin, and independently evaluated by two pathologists.

Immunoreactivity was scored according to the immunoreactive cell percentage and staining intensity on each slide in each low power field (three 100× microscopic fields were randomly selected for each slide). The following staining index was used: 0, tissue with no staining; 1, tissue with faint or moderate staining in <25% of tumor cells; 2, tissue with moderate or strong staining in 25–75% of tumor cells; 3, tissue with strong staining in >75% of tumor cells. Staining intensity was defined as follows: 0, colorless; 1, cream-colored; 2, brown-yellow; 3, tan. The mean product of these two indexes from three fields was defined as the final score: 0–2 (-), 3–4 (+), 5–7 (++), and 8–9 (+++).

### Cell lines and culture

Human colon adenocarcinoma cell line SW480, LoVo, HCT-116, Caco2 and SW620 were obtained from the Central Laboratory of Medical College of Xi’an Jiaotong University. Cell lines SW480, LoVo, HCT-116 and SW620 were cultured in RPMI 1640 medium (Gibco, CA, USA), and the Caco2 cell line was cultured in MEM medium (Gibco, CA, USA) with 10% fetal bovine serum (FBS; Gibco, CA, USA) at 37°C in a humidified incubator with 5% CO_2_. Next, 0.25% of pancreatin (Hyclone, Utah, USA) with 0.05% ethylenediaminetetraacetic acid (EDTA) was used to detach cells for subculture. Drug resistant human colon carcinoma SW480/5-FU and LoVo/5-FU cell lines were purchased from Keygen Biotech (Nanjing, China), and cultured in MEM medium (Gibco, CA, USA), containing 25 μg/ml of 5-FU (Sigma, St. Louis, USA) and 10% FBS (pH 7.4), under the same conditions as the SW480 cell line.

### CAC1-shRNA and stable transfection

The *CAC1-shRNA* and *control-shRNA* were chemically synthesized by Genechem (Shanghai, China), and used for transfection into the colon carcinoma cell lines. The shRNA sequences were as follows: CAC1-shRNA (shCAC1) (forward 5’-GGAUGGUGCCAUAGAUCAATT-3’; reverse 5’-UUGAUCUAUGGCACCAUCCGG-3’), and control-shRNA (shCON) (forward 5’-UUCUCCGAACGUGUCACGUTT-3’; reverse 5’-ACGUGACACGUU CGGAGA ATT-3’).

SW480 cells or SW480/5-FU cells were grown on six-well plates for 24 hours prior to transfection. On the following day, cells were transfected with *CAC1-shRNA* or *control-shRNA* using Lipofectamine 2000 (Invitrogen, NY, USA), according to manufacturer’s instructions. After one week of culture with 2 ng/ml of puromycin, quantitative RT-PCR and western blot were used to evaluate the knockdown efficiency. Then, clones were screened for cells with downregulated CAC1. These clones were named SW480-shCAC1 and SW480/5FU-shCAC1, respectively, and were used for in the subsequent assays. LoVo-shCAC1 and LoVo/5-FU-shCAC1 were also obtained with the above methods.

### Flow cytometry assay

Flow cytometry assay was performed to detect cell cycle distribution and apoptosis. Cells (1×10^5^ cells/well) were cultured in six-well plates for 24 hours and harvested. Then, cells were washed with ice cold phosphate buffered saline (PBS) twice and fixed in 75% ice cold ethanol for two hours at 4°C. Finally, the fixed cells were stained with propidium iodide (PI) containing RNase A at 37°C for 30 minutes. The percentage of cells in each stage of the cell cycle was determined using a FAC sorter (BD, Franklin Lakes, USA), and was calculated using the Cell Quest software (BD, Franklin Lakes, USA).

In order to evaluate the apoptosis, cells were collected and washed, as previously described, for the detection of cell cycle distribution. Next, cells were resuspended with binding buffer at a concentration of 1×10^6^ cells/ml, and stained with Annexin V-FITC and PI. Then, the apoptotic rate was determined and calculated using the FAC sorter and Cell Quest software (BD, Franklin Lakes, USA).

### Quantitative RT- PCR

Total RNA was isolated from cells using Trizol reagent (Invitrogen, Carlsad, USA), according to manufacturer’s protocol, and quantified by spectrophotometry (ND, Wilmington, USA). Next, reverse transcription was performed using a Prime Script RT Reagent Kit (TaKara, Dalian, China). The Premix Ex Taq^™^ II (TaKara, USA) was used to perform the real-time PCR. The oligonucleotide primers for CAC1 and β-actin were designed and synthesized by TaKaRa. The primer sequences were as follows: CAC1 (forward 5’-GCAGCATATTCAGAAAGTTCAGA-3’; reverse 5’-CATTTACAGCCTAATGCCTTTACT-3’), and β-actin (forward 5’-TGGCACCCAGCACAATGAA-3’; reverse 5’-CTAAGTCATAGTCCGCCTAGAAGCA-3’).

The real-time PCR reactions were carried out using an iQ multicolor real-time PCR detection system (Bio-Rad, USA). The relative mRNA expression levels of the target genes were calculated using the ^ΔΔ^Ct method, and β-actin was used as an internal reference gene.

### Western blot

For the western blot analysis, proteins were extracted using RIPA lysis buffer (Roche, Basel, Switzerland). Then, the protein lysates were collected and quantified after incubation on ice and centrifugation (12,000 × g). Next, equal amounts of protein lysates were resolved on 10% sodium dodecyl sulfate-polyacrylamide gel electrophoresis (SDS-PAGE), transferred onto a polyvinylidene fluoride (PVDF) membrane (Millipore, Billerica, USA), and immunoblotted with an antibody against CAC1 (1:500; GeneTex, Texas, USA; Cat no. GTX118514), P-glycoprotein (1:1,000; Santa Cruz, CA, USA), MRP-1 (1:1,000; Santa Cruz, CA, USA) and β-actin (1:5,000; Santa Cruz, CA, USA) overnight at 4°C. On the following day, horseradish peroxidase (HRP)-conjugated secondary antibody (anti-rabbit, 1:2,000; anti-mouse, 1:10,000; Santa Cruz, CA, USA) was added for two hours at 37°C. Finally, the proteins were visualized using enhanced chemiluminescence (ECL; Millipore, Massachusetts, USA) and exposed to X-ray films.

### Colony formation assay

For the colony formation assay, cells (10^3^) were seeded onto 60-mm dishes in triplicate. After 14 days of incubation, the colonies were fixed with methanol and stained with Giemsa. The number of colonies of 10 or more cells was counted under a microscope.

### Cell migration assay

For the migration assay, cells were cultured with serum-free medium at 24 hours prior to the experiment. The cell migration analysis was performed using Transwell plates (Millipore, MA, USA). In brief, 5×10^3^ cells were seeded on the top chamber with medium containing 1% FBS, while a medium with 10% FBS was added to the lower chamber, which was used as a chemoattractant. Then, cells were allowed to migrate through 8-μm pores for 48 hours. Next, cells on the upper surface were removed, and cells that migrated to the bottom side of the membrane were fixed and stained with 0.1% crystal violet solution (Sigma, St. Louis, USA). Finally, cells were counted in 10 fields under a light microscope. All experiments were performed in triplicates.

### Cell invasion assay

For the cell invasion assay, 5×10^3^ cells were seeded in triplicate in growth factor-reduced Matrigel invasion chambers (BD, MA, USA) with serum-free medium. Then, cells were allowed to invade through the Matrigel and 8-μm pores, and towards the medium containing 10% FBS for 48 hours. Non-invading cells were removed, while cells that invaded through the membrane were fixed with ice-cold methanol, stained with 0.1% crystal violet solution, and photographed (at least four fields per membrane). Then, the average number of cells per microscopic field was determined.

### Tumorigenicity assays in nude mice

In order to assess the effects of CAC1 in CRC *in vivo*, a nude mice xenograft assay was performed. Twenty-four 4-6-week-old healthy nude mice (BALB/C, Nu/Nu) were obtained from the Animal Center of the Medical College of Xi’an Jiaotong University, and were randomly allocated into four groups (*n* = 6 mice per group) for treatment with different cells: SW480-shCON, SW480/5FU-shCON, SW480-shCAC1, and SW480/5FU-shCAC1. Then, these cells (1×10^7^) were trypsinized, suspended in 100 μl of PBS, mixed with an equal volume of Matrigel (BD, Franklin Lakes, USA), and inoculated by subcutaneous injection into the right flank of each nude mouse. Tumor growth was monitored by measuring the length (L) and width (W) of the tumor using calipers every seven days, and the tumor volume was calculated using the following formula: tumor volume = 1/2 (L×W^2^). After six weeks of observation, these nude mice were sacrificed, and the tumor xenografts were removed, weighted and photographed. The present study implemented the standard animal handling and experimental procedures, and was approved by the Animal Care and Use Committee of Xi’an Jiaotong University (No. XJTULAC 2017–054).

### Tumor liver metastasis nude mice model

In order to detect the role of CAC1 in invasion and migration *in vivo*, an experimental liver metastasis model was established by injecting cells into the spleens of nude mice. Briefly, the spleen was exposed by an incision in the left upper abdomen, and cells (5×10^5^) in 200 μl of PBS mixed with Matrigel were injected into the spleens of nude mice. After two weeks, these nude mice were euthanized by CO_2_ gas asphyxiation, and the livers were removed and photographed for metastases.

### Statistical analysis

Data were presented as mean ± standard deviation (SD) from at least three independent experiments. Student’s *t*-test was performed for two groups, and the statistical significance was evaluated using one-way ANOVA for multiple groups. A *P*-value of <0.05 was considered statistically significant. *Kaplan-Meier* curves were used to characterize the overall survival (OS) of all patients, and the distribution difference of OS for patients with different CAC1 expression was tested by *log-rank* test. Statistical analyses were performed using SPSS version 13.0 software package (SPSS, Chicago, USA). In each figure, the error bars represent the standard error of the mean, while the statistical significance levels were noted as *P<0.05 and **P<0.01.

## Results

### CAC1 expression in CRC tissue specimens and cell lines

**Fig 1:**
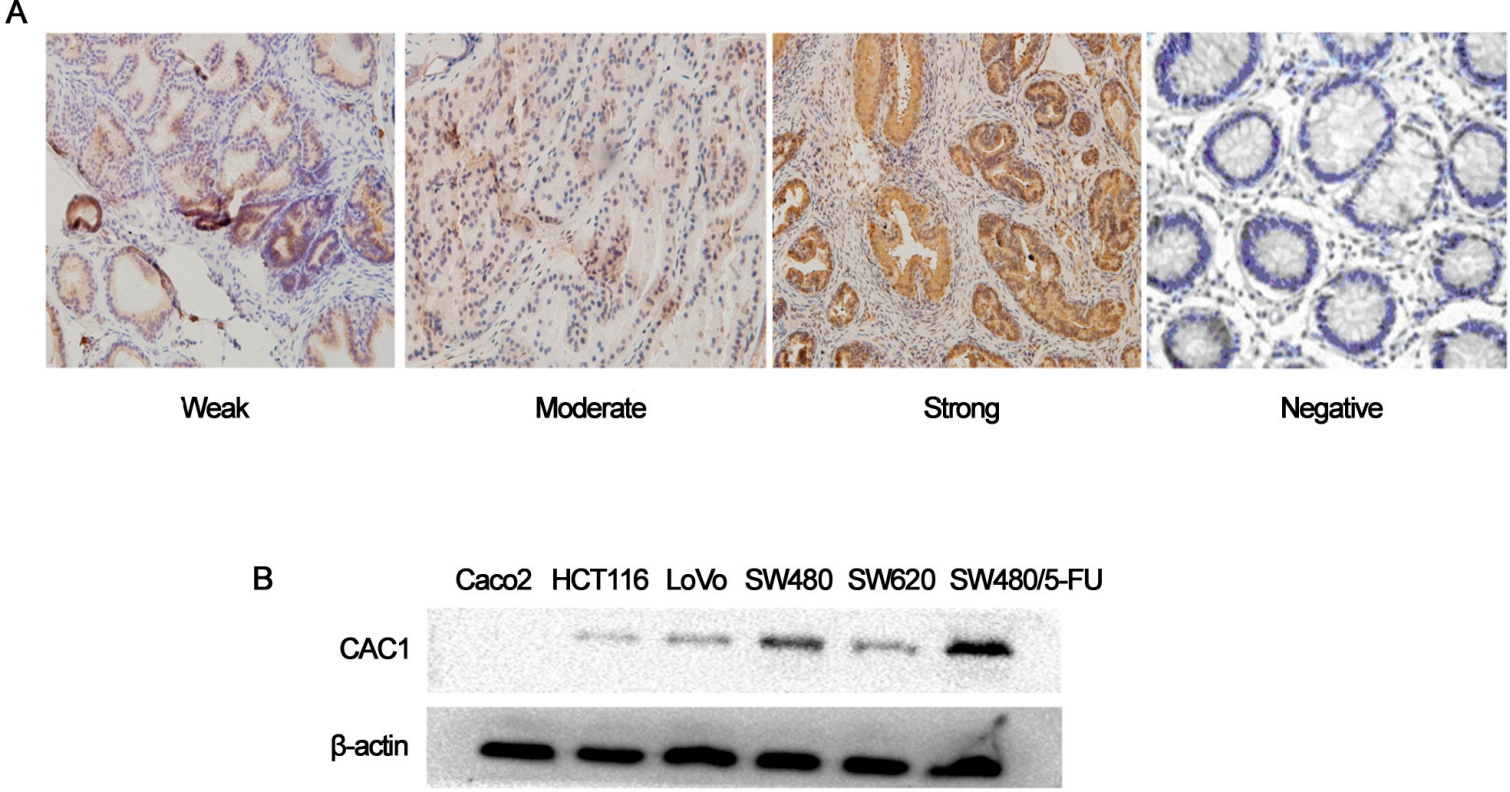
CAC1 protein expression in CRC tissues and cell lines. (A) The immunohistochemistry analysis of CRC tissues and its corresponding normal tissue samples are shown, and the tissue microarray sections were classified into four groups, according to staining intensity (×200). (B) The western blot analysis of CAC1 expression in CRC cell lines and drug resistance cell line SW480/5-FU is shown.

In the present study, immunohistochemistry was used to detect the CAC1 protein expression in CRC specimens obtained from 83 patients and the corresponding normal tissues obtained from the same patient. CAC1 protein was mainly expressed in the cytoplasm, and positive staining was visible as a yellow-brown cytoplasmic staining. In the present study, positive CAC1 immunostaining was observed in 78.3% (65/83) of tumor tissues and 20.5% (17/83) of normal tissue samples (*X*^*2*^ = 55.526, *P*<0.001; Fig 1A). In addition, significant differences in CAC1 protein staining were observed according to the TNM classification (*P*<0.05) and lymph nodes metastasis (*P*<0.05). However, other clinicopathological features of patients and their tumors did not correlate with the expression of CAC1 proteins (*P*>0.05) (Table 1). Western blot was performed to determine the protein expression of CAC1 in the CRC cell lines. The data revealed a significantly higher CAC1 protein expression (*P*<0.05) in SW480 cells, compared to other CRC cell lines (Caco2, LoVo, HCT-116 and SW620), as previously reported. Furthermore, CAC1 protein expression was higher in SW480/5-FU cells, when compared to SW480 cells (Fig 1B), suggesting that CAC1 might be involved in drug resistance in CRC.

### Colorectal cancer patients with CAC1 negative expression have a better prognosis

**Fig 2:**
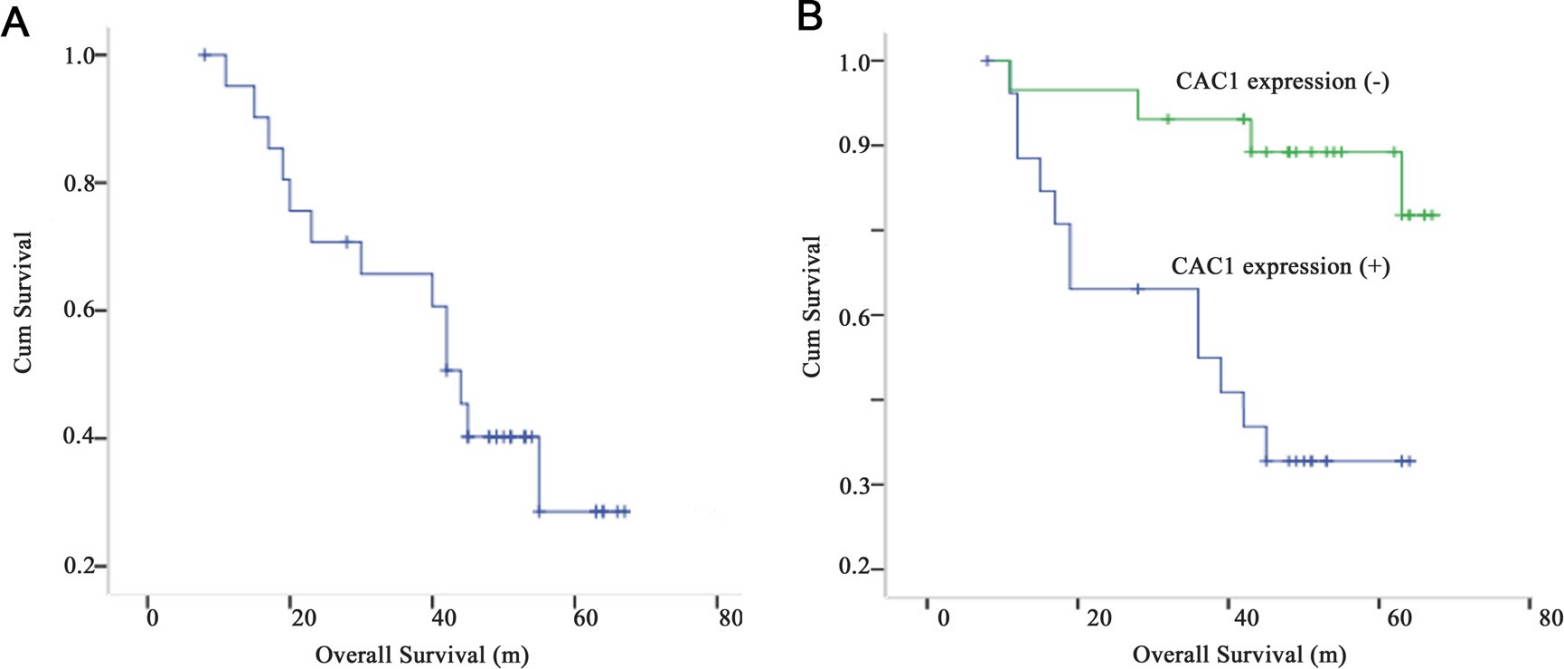
The overall survival curve of colorectal cancer patients with different CAC1 expression. (A) The overall survival curve of the 83 enrolled patients. (B) The overall survival curves of patients with CAC1 positive expression and CAC1 negative expression.

The data up to the cutoff date of December 2013 was used, with a median follow-up time of 42 months (range: 6–63 months). The OS of the enrolled 83 participants ranged within 6–63 months, with a median OS of 42 months. The overall survival curve is presented in Fig 2A. The Kaplan-Meier estimate of the 5-year survival rate was 27.65%. A total of 67 patients were included in the CAC1 positive expression group, while 16 patients were included in the negative expression group. The median OS was 24 months (range: 6–60 months) in the CAC1 positive group *vs*. 46 months (range: 10–63 months) in the CAC1 negative group (*P*<0.05). The 5-year OS rate was 34.22% in the CAC1 positive expression group and 76.4% in the other group (*P*<0.01, Fig 2B).

The subgroup analyses revealed that the prognosis of patients with negative CAC1 expression was better than the prognosis of CAC1 positive patients. Based on these findings, it can concluded that CAC1 affects tumor progression, and consequently, patient survival.

### CAC1 knockdown induced the arrest of 5-FU resistant cells in the G1/S phase

**Fig 3:**
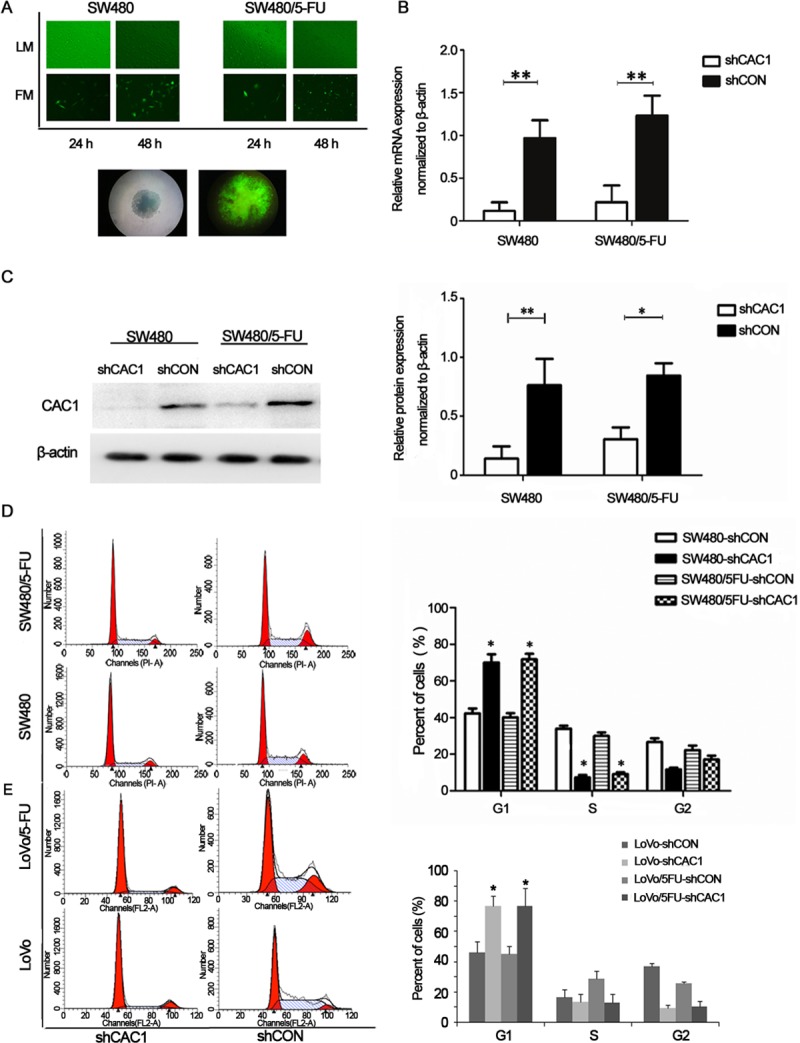
CAC1 knockdown induced the arrest of SW480/5-FU cells in the G1/S phase. (A) The stable shCAC1 transfection of SW480 and SW480/5-FU cells for 24–48 hours. (B) The quantitative RT-PCR analysis of CAC1 mRNA expression in SW480 and SW480/5-FU cells following CAC1 knockdown. (C) The western blot analysis of CAC1 protein expression in SW480 and SW480/5-FU cells following CAC1 knockdown. CAC1 knockdown induced cell cycle arrest at the G1 phase in SW480, SW480/5-FU cells (D) and LoVo, LoVo/5-FU cells (E). Cells were stably transfected with shCAC1 for one week, and the cell cycle profile was detected using FACS analysis following propidium iodide staining. The representative results of three independent experiments are shown. *P<0.05, **P<0.01.

In order to assess the role of CAC1 in CRC drug resistance, CAC1 expression was knocked down in SW480 and SW480/5-FU cells through the stable transfection with CAC1-shRNA (Fig 3A). The efficiency of CAC1 knockdown in SW480-shCAC1 and SW480/5FU-shCAC1 cells was confirmed at both the protein and mRNA level (Fig 3B and 3C).

Cell cycle distributions were determined by flow cytometry assay in SW480 cells, SW480/5-FU cells, and the corresponding shCAC1 transfected cells. The data revealed that the proportion of SW480-shCAC1 cells and SW480-shCON cells at the G1/S phase was 70.02 ± 4.50% and 42.29 ± 2.70%, respectively, and the difference was statistically significant (*P*<0.05). The percentage of sub-G1 fraction in SW480/5FU-shCAC1 cells (71.80 ± 2.97%) was remarkably higher, when compared to the SW480/5FU-shCON cells arrested at the G1 phase (40.13 ± 2.25%) (*P*<0.05, Fig 3D) (data in S1 Dataset). There was 76.98 ± 11.54% of cells arrested at the sub-G1 stage in LoVo/5FU-shCAC1 cells, and 45.3 ± 5.4% of cells in LoVo/5-FU cells (*P*<0.05, Fig 3E) (data in S1 Dataset). SW480, LoVo, and their corresponding 5-FU resistant cells were all arrested at the G1/S phase of the cell cycle after CAC1 knockdown. Collectively, these data indicate that CAC1 is involved in the control of critical G1/S machinery transition, and that this promotes G1-S transition in both parental cells and 5-FU resistant cells.

### CAC1 knockdown induced cell apoptosis and increased the sensitivity of 5-FU resistant cells to 5-FU by influencing P-glycoprotein and MRP-1 expression

**Fig 4:**
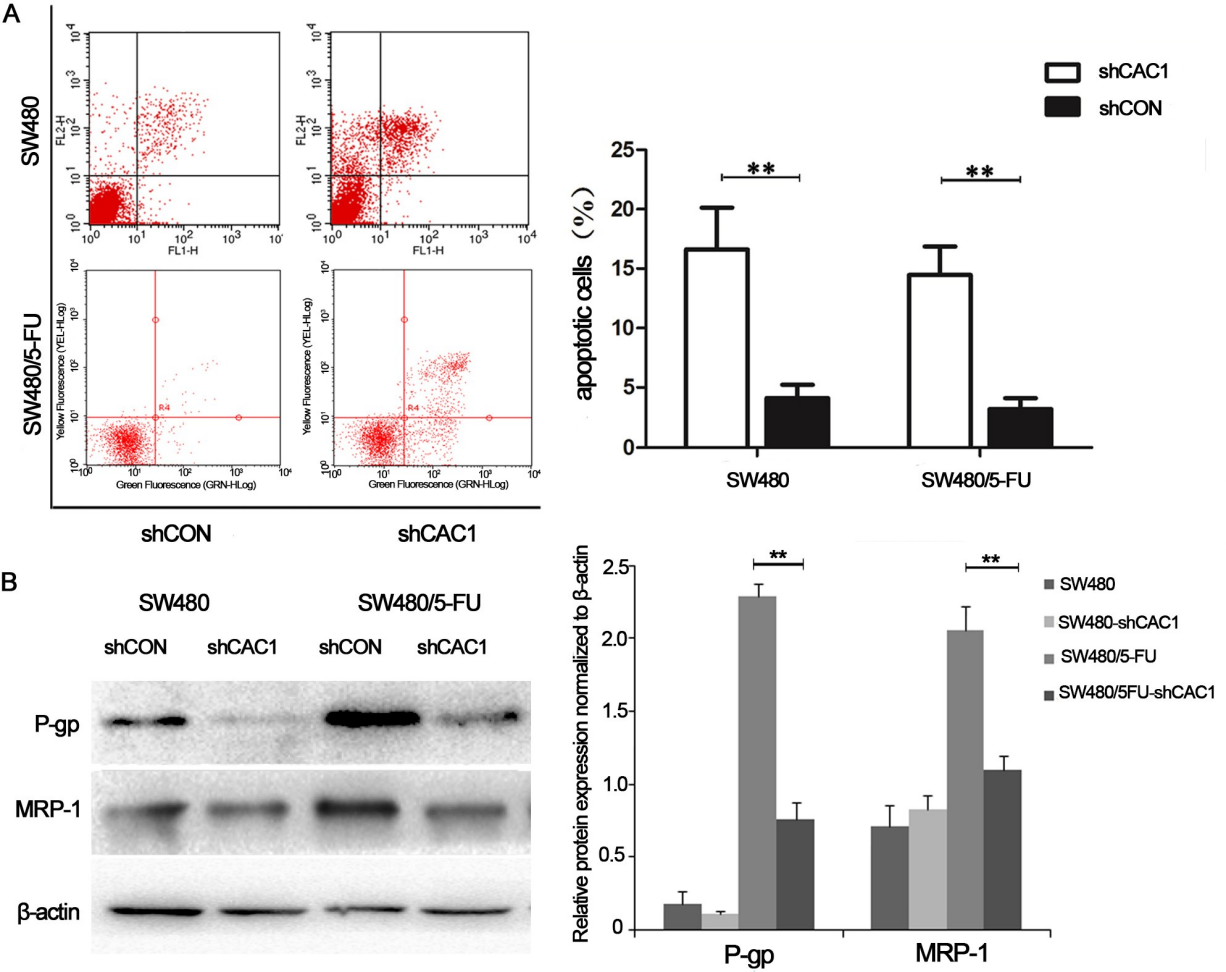
CAC1 knockdown increased sensitivity of SW480/5-FU cells to 5-FU by influencing P-glycoprotein and MRP-1 expression. (A) SW480 and SW480/5-FU cells were transfected with shCAC1 and shCON for one week, and subjected to FACS analysis with Annexin V-FITC and propidium iodide staining. (B) The expression of drug resistance-associated proteins, P-glycoprotein and MRP-1, in SW480 and SW480/5-FU cells after transfection with shCAC1 and shCON was examined by western blot. *P<0.05, **P<0.01.

**Fig 5:**
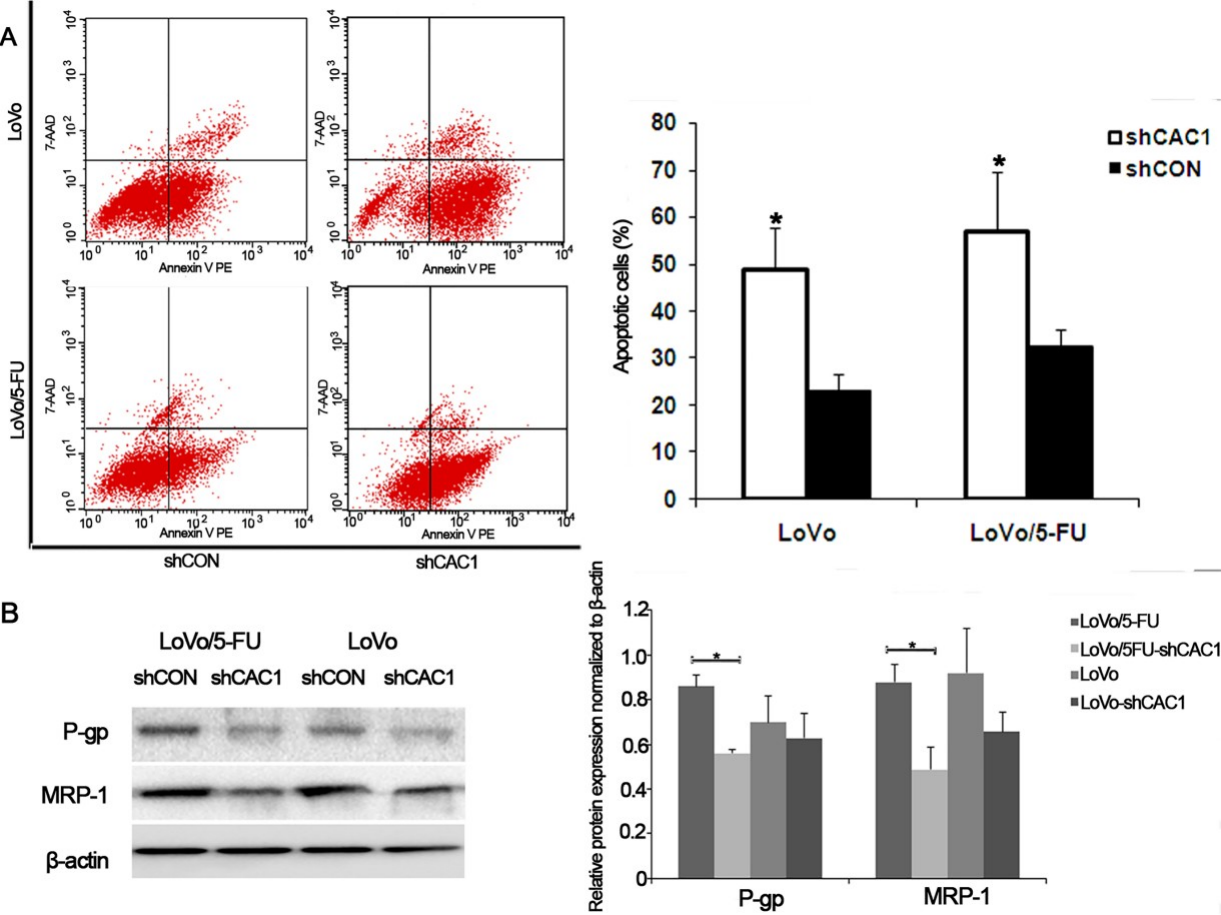
CAC1 knockdown increased sensitivity of LoVo/5-FU cells to 5-FU by influencing P-glycoprotein and MRP-1 expression. (A) LoVo and LoVo/5-FU cells transfected with shCAC1 and shCON were subjected to FACS analysis with Annexin V-FITC and propidium iodide staining. (B) The expression of drug resistance-associated proteins, P-glycoprotein and MRP-1, in LoVo and LoVo/5-FU cells after transfection with shCAC1 and shCON was examined by western blot. *P<0.05, **P<0.01.

The measurement of apoptosis was carried out by flow cytometry with Annexin-V/PI staining. The results revealed an increase in apoptotic SW480 cells following CAC1 knockdown (*P*<0.05, Fig 4A). In SW480-shCON cells, 4.10 ± 1.12% of apoptotic cells were detected, while after the knockdown of CAC1, 16.62 ± 3.51% of apoptotic cells were detected in SW480-shCAC1 cells. There were 22.96 ± 3.55% and 48.66 ± 9.20% of apoptotic cells in the LoVo-shCON and LoVo/5FU-shCAC1 sub-groups, respectively (*P*<0.05, Fig 5A) (data in S1 Dataset). Based on these results, it appears that CAC1 is involved in the regulation of apoptosis in CRC cells, and that CAC1 knockdown could enhance CRC cell apoptosis.

Next, CAC1 silencing was evaluated to determine whether this may modulate 5-FU-induced apoptosis. For this purpose, SW480/5FU-shCON and SW480/5FU-shCAC1 cells were treated with 5-FU. The annexin-V/PI staining revealed a slighty higher increase in cell apoptosis in SW480/5FU-shCAC1 cells (14.47 ± 2.39%), when compared to SW480/5-FU cells transfected with shCON (3.17 ± 0.92%) (*P*<0.05, Fig 4A). The apoptotic cells in LoVo/5FU-shCAC1 cells (56.89 ± 12.63%) were higher than those in LoVo/5-FU cells (32.26 ± 6.94%) (*P*<0.05, Fig 5A). Collectively, these findings indicate that CAC1 is involved in the resistance to chemotherapy, and that its silencing could increase the sensitivity of CRC cells to 5-FU.

In order to further examine the possible mechanism of the influence of CAC1 in CRC drug resistance, western blot analysis was performed. Indeed, it was found that CAC1 could affect the expression of P-glycoprotein and MRP-1 in the 5-FU resistant cell line. With the condition of equal β-actin expression, P-glycoprotein and MRP-1 significantly decreased in SW480/5FU-shCAC1 and LoVo/5FU-shCA1 cells (Figs 4B and 5B). In SW480 cells, P-glycoprotein expression decreased and MRP-1 protein accumulated following CAC1 knockdown In LoVo cells, P-glycoprotein and MRP-1 both decreased following CAC1 knockdown. Based on these results, it can be concluded that CAC1 could be involved in drug resistance in CRC cells.

### Effects of CAC1 knockdown on CRC cell colony formation and invasive and migration ability *in vitro*

**Fig 6:**
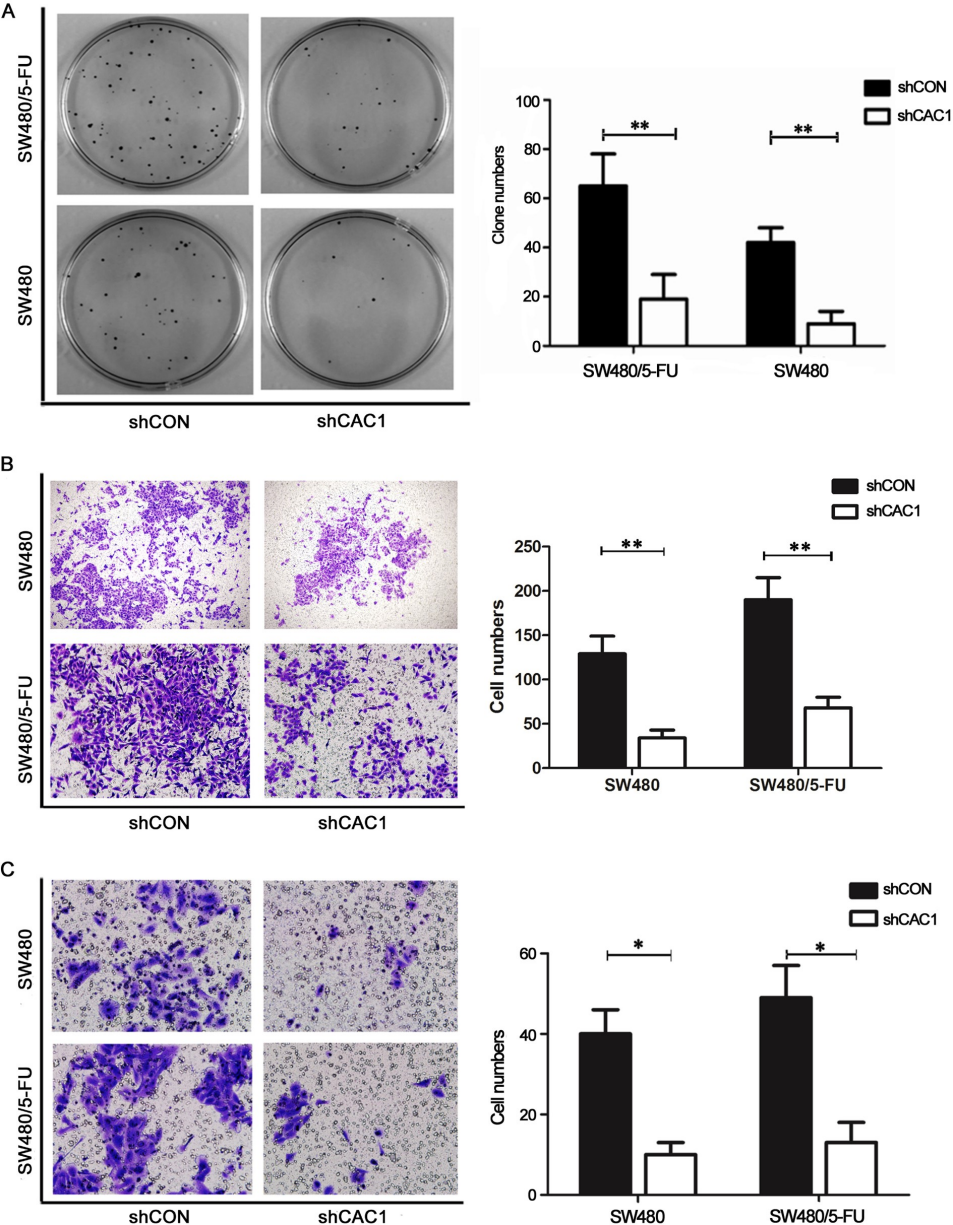
CAC1 knockdown reduced colony formation and invasive and migration ability of SW480 and SW480/5-FU cells. (A) Colony formation assay, (B) cell migration assay and (C) cell invasion assay were detected in SW480 and SW480/5-FU cells were stably transfected with shCAC1 and shCON for one week. *P<0.05, **P<0.01.

**Fig 7:**
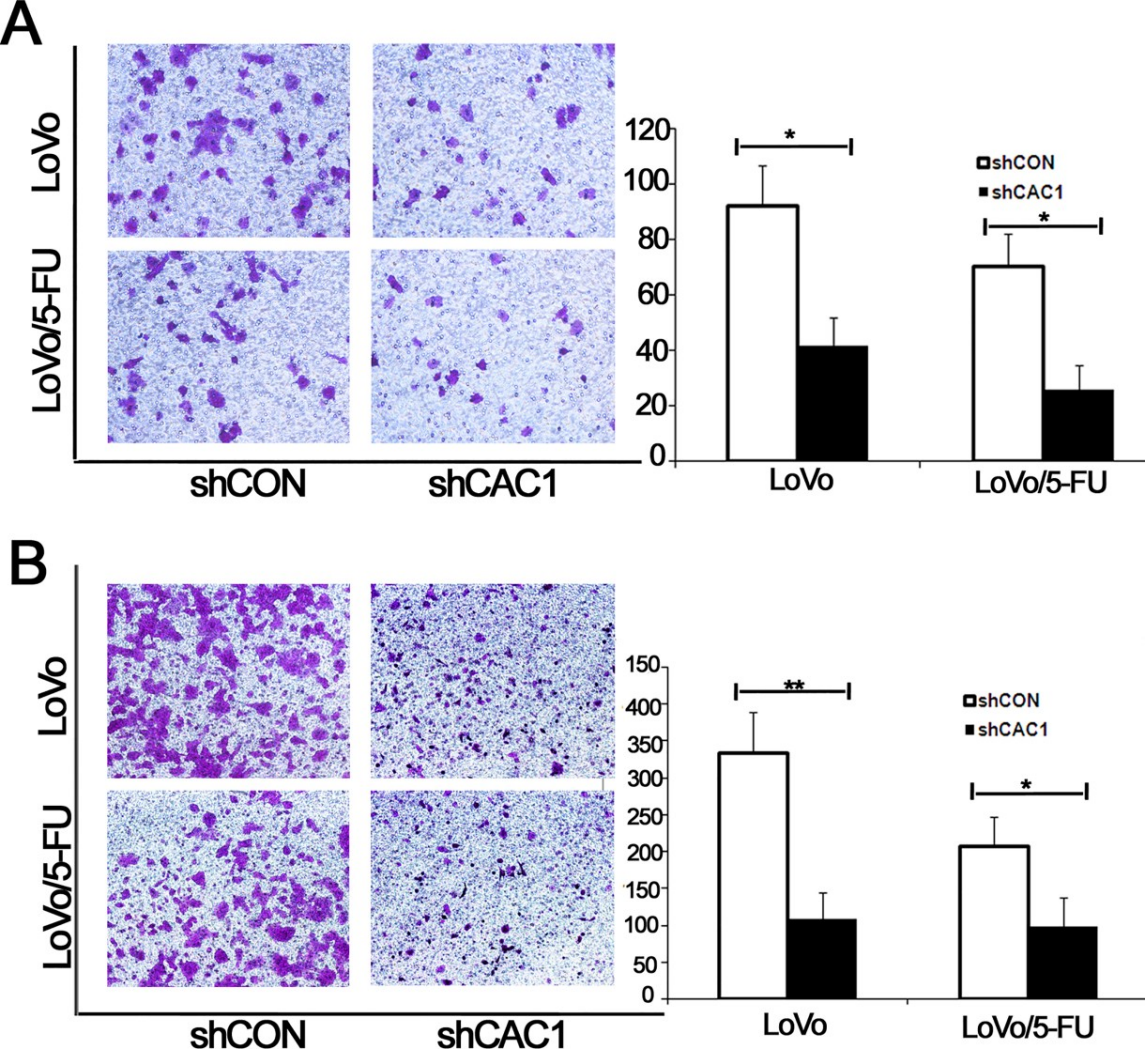
CAC1 knockdown reduced the invasive and migration ability of LoVo and LoVo/5-FU cells. (A) Cell invasion assay and (B) cell migration assay were detected in LoVo and LoVo/5-FU cells transfected with shCAC1 and shCON, respectively. *P<0.05, **P<0.01.

Next, the effects of CAC1 silencing in SW480 and SW480/5-FU cells were evaluated. The colony formation assay revealed that the number of colonies that formed in cells, in which CAC1 was downregulated, sharply decreased, when compared with SW480 and SW480/5-FU cells transfected with shCON (*P*<0.05). In addition, the size of colonies that formed from the shCAC1 transfected cells was more significant (Fig 6A). Furthermore, compared to SW480 and SW480/5-FU cells, both invasive and migration ability were lower in cells transfected with shCAC1 (Fig 6B and 6C) (data in S1 Dataset). The same trends were also observed in LoVo and LoVo/5-FU cells (Fig 7) (data in S1 Dataset).

### CAC1 knockdown reduced tumorigenicity and metastasis and downregulated P-glycoprotein and MRP-1 expression *in vivo*

**Fig 8:**
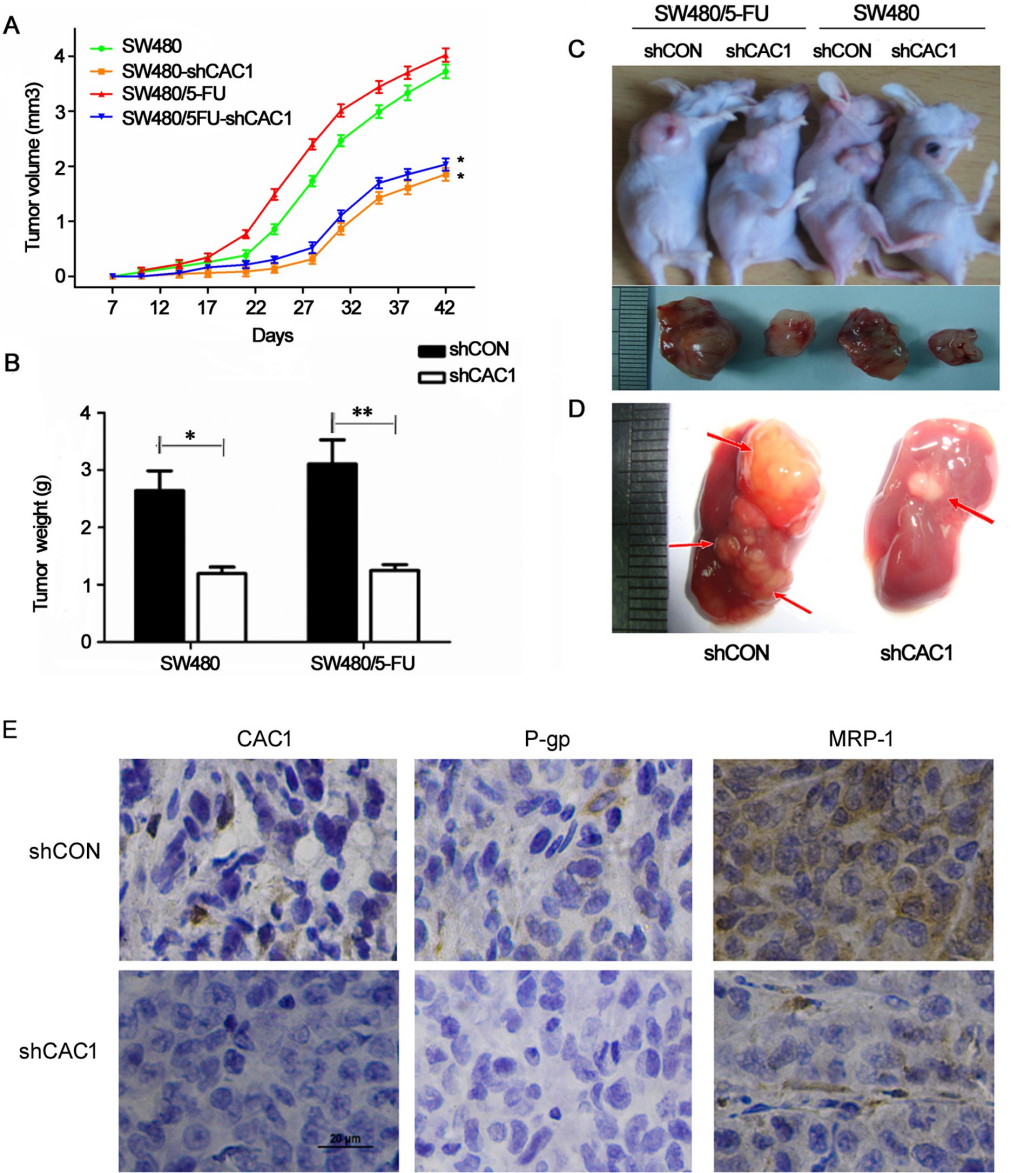
CAC1 knockdown reduced the growth of SW480/5-FU cell xenografts and liver metastatic lesions *in vivo*. (A) Tumor growth curve and (B) tumor weight in each group. (C) Representative photographs of tumors and (D) liver metastatic lesions isolated from nude mice in each group at six weeks after inoculation with SW480-shCON, SW480-shCAC1, SW480/5FU-shCON and SW480/5FU -shCAC1. (E) The levels of CAC1, P-gp and MRP-1 in transplanted tumors were detected by IHC. The expression of CAC1, P-gp and MRP-1 was lower in SW480/5FU-shCAC1 transplanted tumors, when compared to the SW480/5FU-shCON group. *P<0.05, **P<0.01.

In order to further investigate the effects of CAC1 knockdown *in vivo*, nude mice xenografts were established. As shown in Fig 8A–8C, SW480/5FU-shCON xenograft tumor volume and weight were larger and heavier than SW480/5-FU xenografts transfected with shCAC1 (*P*<0.05) (data in S1 Dataset). The same trend was observed for SW480-shCON and SW480-shCAC1 cell xenografts. As shown in Fig 8D, the number of liver metastatic lesions in SW480/5FU-shCON mice was higher, and the observed volume of lesions was also larger than in SW480/5FU-shCAC1 mice. Furthermore, immunohistochemical analysis was performed in transplanted tumors derived from SW480/5FU-shCON and SW480/5FU-shCAC1 groups and CAC1 knockdown downregulated P-glycoprotein and MRP-1 expression *in vivo* (Fig 8E). Based on these findings, it can be concluded that CAC1 has a potential role in promoting tumorigenicity, accelerating tumor metastasis and reversing drug resistance *in vivo*.

## Discussion

CAC1 is a member of the cullin family, and has been reported to be overexpressed in various cancers such as breast [16], lung [22] and gastric cancer [23]. In recent years, it has been shown that CAC1 promotes cell cycle progression in some cancer cell lines [11, 22, 23]. However, few studies have examined its possible role in drug resistance in human CRC. It has been reported that CAC1 may be involved in drug resistance in CRC through targeting miR-199a-5p [24]. In the present study, CAC1 proteins were reported to be overexpressed in 78.3% of CRC specimens and 20.5% of normal tissue samples. In addition, a significant difference in CAC1 protein expression was observed according to TNM stage and lymph node metastasis. Among the examined CRC cell lines, CAC1 expression was the highest in the SW480 cell line, and its expression was even higher in its drug resistant counterpart SW480/5-FU cell line. In the present study, CAC1 knockdown induced cell cycle arrest at the G1/S phase and apoptosis in 5-FU resistant cells. The sensitivity to 5-FU possibly increased by regulating P-glycoprotein and MRP-1 protein expression. Furthermore, their invasive and migration ability declined in cells after CAC1 silencing. Nude mice xenograft analyses further confirmed that CAC1 knockdown reduced tumorigenicity and metastasis *in vivo*.

Previous studies have shown that CAC1 has an oncogene-like function related to cell cycle regulation [11, 17, 23], and the present results strongly confirm these findings. The present study on CAC1 was capable of promoting cell cycle progression at the G1/S phase transition in SW480, LoVo, and the corresponding 5-FU resistant cells. Although the mechanism on how CAC1 regulates the cell cycle was not determined in the present study, CDK2 protein and cyclin E have already been suggested to participate in these processes in other correlation studies [11, 23, 24]. Based on these findings, it can be concluded that CAC1 plays an important role in drug resistance by inducing cell cycle arrest.

The role of CAC1 in cell cycle regulation raised the possibility of its participation in cell apoptosis. The induction of cell apoptosis is regarded as an important tumor suppressive mechanism [25]. As expected, the rate of early apoptotic SW480-shCAC1 cells was significantly higher than the apoptotic rate observed in SW480 control cells. The same effect of CAC1 was observed in the LoVo cell line. Thus, in the present study, the CAC1 expression in SW480 cells protected them from apoptosis. Nevertheless, the exact role of CAC1 in cell apoptosis remains not fully understood. It has been suggested that CAC1 knockdown might lead to increased cellular stress response and p53 protein expression [17]. In another study, CAC1 was suggested as the positive regulator of Nrf2 in cell survival [12].

The 5-FU remains an important agent in oncological treatment of CRC. It acts as a kind of antimetabolite that has a cytotoxic effect, induces DNA damage, and subsequently induces tumor cell apoptosis [26]. Although 5-FU has been shown to have advantages in cancer chemotherapy, the gains are relatively modest, and the benefits are obtained at the cost of the morbidity of patients [10]. In addition, drug resistance brings new challenges to chemotherapy for cancer patients. Indeed, the development of drug resistance is a critical problem that has decreased the number of CRC patients who remain in long-term remission [27]. In the present study, it has been shown that CAC1 expression is associated with drug resistance in CRC. The 5-FU treatment resulted in a remarkable increase in apoptotic cells in SW480/5FU-shCAC1 and LoVo/5FU-shCAC1 cells. CDK2 has been proven to be involved in acquired 5-FU resistance in colon cancer [18], and that it could regulate the ABC transporter [19]. Since CAC1 regulates CDK2 activity, it is possible that CAC1 can reverse 5-FU resistance by regulating the ABC transporter. In order to further explore this possibility, the expression of P-glycoprotein and MRP-1, were examined. Furthermore, it was found that these were both downregulated when CAC1 was silenced. It has been previously shown that bax and p53 proteins are upregulated and bcl-2 proteins are downregulated when CAC1 knockdown was carried out in cisplatin-induced apoptosis AGS gastric cancer cells [23]. Furthermore, Kong *et al*. [24] reported that CAC1 is involved in CRC multi-drug resistance through the upregulation of MDR1 expression.

With the use of an *in vitro* CRC drug resistance cell model, the cancer-related phenotypes of tumor cells, including colony formation, invasion and migration, were evaluated. In the present study, 5-FU resistant cells exhibited a significantly greater ability to migrate and invade, and when CAC1 was silenced, this phenotype sharply diminished. It has been previously shown in gastric cancer that *Helicobacter pylori* promotes gastric cancer cell invasion and metastasis through the activation of AP-1 and the upregulation of CAC1 [28], suggesting that CAC1 is involved in the invasion and metastasis of gastric cancer. It is possible that CAC1 may affect the ability of cancer cells to migrate and invade through the influence it exerts on miR-139-5p, which has been previously reported [24]. Indeed, in a study conducted by Zhang *et al*., it was found that miR-139-5p was the most downregulated miRNA in CRC [29]. Furthermore, the study conducted by Shen *et al*. has proven that miR-139-5p is a metastatic suppressor in CRC [30].

Next, the investigators examined the role of CAC1 in tumorigenicity and metastasis *in vivo* in a nude mice xenograft and liver metastasis model. In SW480/5-FU cells with silenced CAC1 expression, the tumor volume and weight were smaller, and a longer tumor formation time was observed, when compared with SW480/5FU-shCON mice. In addition, there were more liver metastatic lesions, which were also larger in the SW480/5-FU group, when compared to mice with SW480/5FU-shCAC1 xenografts. The same phenomenon was observed when xenografts of SW480-shCON cells and SW480-shCAC1 cells were examined. These experiments revealed that the *in vitro* and *in vivo* findings on the role of CAC1 were consistent. Collectively, the results obtained *in vivo* strongly suggest that CAC1 expression is intimately involved with CRC progression, metastasis and drug resistance.

In conclusion, the present study has shown that CAC1 plays a pivotal role in colorectal tumor progression and drug resistance. Furthermore, the present data suggests that CAC1 may be used as a potential target for the development of new treatments in human CRC with drug resistance. Nevertheless, further studies clarifying the detailed mechanism of CAC1 involvement in CRC drug resistance are needed to validate the present results. Drug resistance is the most significant reason for the failure of chemotherapy. Once drug resistance is acquired, the effect of chemotherapeutic drugs is decreased. The potential mechanisms of drug resistance are mainly attributed to apoptosis/autophagy induction, cancer stem cells, miRNAs, hypoxia, DNA damage and repair, and even epigenetic regulation. CAC1, as a well-known cell cycle modulator, has been proven to be involved in drug resistance, which indicates that it could be used as a novel target for reversing drug resistance in the future to improve the outcome of cancer patients. Furthermore, these present findings provide the possibility that cell cycle regulation may be a new drug resistance mechanism in cancers.

## Supporting information

S1 Dataset This is the data of the current manuscript. (XLSX)
